# Effects of increasing levels of benzoic acid fed to pigs on nitrogen utilization and metabolism affecting growth performance, ammonia emissions, and carcass characteristics

**DOI:** 10.1093/jas/skaf101

**Published:** 2025-03-30

**Authors:** Sung Woo Kim, Hyunjun Choi, Carol Lin, Ronald D Mateo

**Affiliations:** Department of Animal Science, North Carolina State University, Raleigh, NC 27695, USA; Department of Animal Science, North Carolina State University, Raleigh, NC 27695, USA; Department of Animal and Food Sciences, Texas Tech University, Lubbock, TX 70828, USA; Department of Animal and Food Sciences, Texas Tech University, Lubbock, TX 70828, USA

**Keywords:** ammonia emission, benzoic acid, carcass characteristics, growth performance, pigs

## Abstract

The objectives of this study were to investigate the effects of increasing levels of benzoic acid (**BA**) on nitrogen utilization and metabolism affecting growth performance, ammonia emissions, and carcass characteristics, and to determine the optimal levels of BA for the growth performance and carcass characteristics when fed to pigs from weaning to market. A total of 480 pigs (6.0 ± 1.5 kg) were assigned to 4 dietary treatments in a randomized complete block design with initial body weight and group as blocks and were fed in 6 phases. Treatments included a basal diet with antibiotics (positive control, **PC**) and diets with 3 levels of BA (0.00%, 0.50%, and 1.00% BA) without antibiotics. The PC increased (*P* < 0.05) the average daily gain (**ADG**) and G:F during the overall period compared to no BA supplementation. Increasing levels of BA increased (*P* < 0.05) overall ADG quadratically (maximum at 0.53% or 7.5 g/d of BA). Increasing levels of BA increased (*P* < 0.05) overall G:F quadratically (maximum at 0.57% or 8.1 g/d of BA). Increasing levels of BA tended to increase (linear, *P* = 0.096) N digestibility and increased (linear, *P* < 0.05) N retention. The BA supplementation at 1.00% decreased (*P* < 0.05) urine pH and aerial ammonia emission from manure compared to no BA supplementation in the 24 h collection period. The BA supplementation at 1.00% decreased (*P* < 0.05) rate of change in aerial ammonia emission compared to no BA supplementation in the 24 h collection period. The PC increased (*P* < 0.05) shrink weight, hot carcass weight, and first rib backfat compared to no BA supplementation. Increasing levels of BA decreased (*P* < 0.05) loin color and marbling score linearly and increased (*P* < 0.05) the loin eye area quadratically (maximum at 0.59% or 8.1 g/d of BA). In conclusion, supplementation of BA in feeds enhanced growth performance, improved N utilization, reduced urine pH, reduced aerial ammonia emissions, and improved carcass characteristics of pigs. Supplementation of BA at a range of 0.53% to 0.59% (corresponding to 7.5 to 8.1 g/d of BA based on overall average daily feed intake) provided the optimal improvements in body weight gain, feed efficiency, and carcass characteristics when fed to pigs from weaning to market.

## Teaser

Supplementing BA in pig feeds at levels between 0.53% and 0.59% improved weight gain, feed efficiency, and loin eye area in pigs from weaning to market. Supplementing BA in pig feed enhanced urine acidification and decreased ammonia emissions from manure.

## Introduction

The widespread use of antibiotics as growth promoters has been phased out in many countries due to concerns about microbial resistance (Casewell et al., 2003; Tiseo et al., 2020), leading to increased demand for antibiotic alternatives in pig feeds (Zheng et al., 2021; Duarte and Kim, 2022). Among these alternatives, benzoic acid (**BA**) has gained attention for its positive effects on the growth performance of pigs (Halas et al., 2010; Chen et al., 2017; Choi et al., 2023). BA, with acidic properties, serves as a feed additive in pig production, enhancing nitrogen (**N**) utilization through its antimicrobial action in the gastrointestinal tract (Kluge et al., 2006). Benzoic acid supplementation also reduces ammonia-producing bacteria such as *Escherichia coli* and lowers ammonia-N content in the gastrointestinal tract of pigs (Diao et al., 2014), potentially decreasing the incidence of postweaning diarrhea in nursery pigs (Silveira et al., 2018).

Once absorbed through the small intestine of pigs (Kristensen et al., 2009), BA is transferred to the liver, where it conjugates with glycine to form hippuric acid (Murphy et al., 2011), which is subsequently excreted in the urine. Hippuric acid in the urine decreases urine pH (Bühler et al., 2006), reducing urease activity and aerial ammonia emissions from manure, thereby lowering environmental pollution from pig production (De Vries and Melse, 2017). These beneficial effects of BA can enhance the growth performance and carcass characteristics of pigs. However, depending on the level of dietary BA, its impacts on growth are not always consistent (Shu et al., 2016). Excessive BA supplementation (over 2.5% up to 5.0%) can reduce the growth performance of nursery pigs (Shu et al., 2016), and a previous study suggests 0.60% would be an optimal BA level for body weight (BW) gain throughout the overall nursery phase (Choi and Kim, 2024). Based on previous findings, it was hypothesized that appropriate BA supplementation would improve N utilization, increase urine acidification via BA metabolism, reduce ammonia emissions, and ultimately enhance the growth performance and carcass characteristics of pigs. To test this hypothesis, the objectives of this study were to investigate the effects of increasing levels of BA on growth performance, N utilization and metabolism, ammonia emissions, and carcass characteristics, and to determine the optimal levels of BA for growth performance and carcass characteristics when fed to pigs from weaning to market.

## Materials and methods

The protocol of this experiment was reviewed and approved by the Institutional Animal Care and Use Committee of North Carolina State University (Raleigh, NC) and Texas Tech University (Lubbock, TX).

### Animals, experimental design, and experimental diets

A total of 480 pigs (Camborough 22 × PIC boar 327), weaned at 21 d of age with an initial body weight (**BW**) of 6.0 ± 1.5 kg, were assigned to 4 dietary treatments in a randomized complete block design, with initial BW and group as blocks. There were 4 replicates (2 barrow pens and 2 gilt pens) per treatment in groups 1 and 2 respectively, and 7 replicates (4 barrow pens and 3 gilt pens) per treatment in group 3, resulting in a total of 15 pens (8 pigs per pen) per treatment. Pigs were housed in pens with free access to feeds and water throughout the experimental period. Treatments included a basal diet with antibiotics (positive control, **PC**) and 3 levels of BA (0.00%, 0.50%, and 1.00% BA) without antibiotics. Antibiotics were supplemented with 100 mg/kg of chlortetracycline (450 mg/kg of Aureomycin 100) in phases 1 through 3 and were supplemented with 44 mg/kg of tylosin (200 mg/kg of Tylan 100) in phases 4 through 6. Antibiotics or BA were supplemented to the basal diet at the expense of a premix supplement containing 50% corn and 50% animal fat (Table 1). The BA used in this study was in free form. Experimental diets for each phase were formulated to meet or exceed the nutrient requirement estimates suggested by NRC (1998) as the experiment was conducted before publication of NRC (2012). Experimental diets were provided in mash form. Pigs were fed experimental diets based on a 6-phase feeding program until they reached 110 kg BW.

**Table 1.** Composition of basal diets (as-fed basis)

Item	Phase 1^1^	Phase 2^2^	Phase 3^3^	Phase 4^4^	Phase 5^5^	Phase 6^6^
Feedstuff, %						
Corn, yellow dent	35.05	43.60	59.65	68.90	76.40	78.60
Soybean meal, dehulled	22.00	29.00	34.00	26.00	19.00	17.00
Dried whey	27.50	17.50	0.00	0.00	0.00	0.00
Fish meal	4.50	0.00	0.00	0.00	0.00	0.00
Animal plasma	4.00	3.00	0.00	0.00	0.00	0.00
Animal fat	2.00	1.50	1.00	1.00	1.00	1.00
Limestone	0.70	0.70	0.70	0.75	0.75	0.60
Dicalcium phosphate	0.40	1.50	1.30	1.10	0.90	0.85
Sodium chloride	0.45	0.35	0.25	0.15	0.15	0.15
Vitamin–mineral premix^7^	2.00	1.50	2.00	1.00	0.70	0.70
Zinc oxide	0.30	0.25	0.00	0.00	0.00	0.00
Supplement^8^	1.10	1.10	1.10	1.10	1.10	1.10
Calculated composition						
Dry matter, %	91.14	90.66	89.92	89.70	89.58	89.53
Metabolizable energy, kcal/kg	3,300	3,289	3,310	3,355	3,375	3,382
Crude protein, %	23.03	22.15	21.19	18.16	15.46	14.69
SID^9^ Lys, %	1.31	1.20	1.03	0.83	0.66	0.61
SID Met+Cys, %	0.69	0.64	0.60	0.53	0.46	0.45
SID Thr, %	0.83	0.76	0.66	0.55	0.46	0.44
SID Trp, %	0.27	0.26	0.23	0.18	0.15	0.14
Ca, %	0.85	0.91	0.78	0.70	0.61	0.54
STTD^10^ P, %	0.51	0.52	0.38	0.33	0.28	0.26

^1^Phase 1 was 1 wk postweaning (21 to 28 d of age).

^2^Phase 2 was 2 wk after phase 1 (29 to 42 d of age).

^3^Phase 3 was 3 wk after phase 2 (43 to 63 d of age).

^4^Phase 4 was from the end of phase 3 to 121 d of age.

^5^Phase 5 was from 122 to 157 d of age.

^6^Phase 6 was from 157 to 171 d of age.

^7^Vitamin–mineral premix provided the following concentrations per kilogram of complete diet: 31.1 mg of manganese as manganous oxide, 50 mg of iron as iron sulfate, 69.2 mg of zinc as zinc oxide, 6.32 mg of copper as copper oxide, 0.48 mg of iodide as ethylenediamine dihydroiodide, 5,037.4 IU of vitamin A as vitamin A acetate, 550 IU of vitamin D_3_, 41.3 IU of vitamin E, 2.9 IU of vitamin K as menadione sodium bisulfite, 36.6 g of vitamin B12, 9.2 mg of riboflavin, 29.3 mg of D-pantothenic acid as calcium pantothenate, 36.6 mg of niacin, and 3,224 mg of choline as choline chloride.

^8^Four dietary treatments were formulated: a PC diet containing antibiotics and diets with 3 levels of BA at 0.00%, 0.50%, and 1.00% BA without antibiotics. In the PC diet, antibiotics were included at 100 mg/kg of chlortetracycline (equivalent to 450 mg/kg of Aureomycin 100) during phases 1 to 3 and 44 mg/kg of tylosin (equivalent to 200 mg/kg of Tylan 100) during phases 4 to 6. Both antibiotics and BA were supplemented by replacing a premix supplement in the basal diets. The premix supplement consisted of 50% corn and 50% animal fat. The total inclusion rate of antibiotics or BA plus the premix supplement was maintained at 1.1% across all dietary treatments, such that increasing the level of BA or antibiotics resulted in a proportional reduction in the amount of the premix supplement.

^9^Standardized ileal digestible. Coefficients were taken from NRC (2012).

^10^Standardized total tract digestible. Coefficients were taken from NRC (2012).

### Growth performance and diarrhea incidence

Pigs were housed in a nursery building during phases 1, 2, and 3, and then the pigs were moved to a grower-finisher building until they completed phase 6, reaching a final BW of an average of 110 kg. The pigs were kept in their respective pens throughout the experiment. The BW of the pigs and feed disappearance per pen were measured at the end of each phase to determine average daily gain (**ADG**), average daily feed intake (**ADFI**), and gain-to-feed ratio (**G:F**) for growth performance. During the first 15-d postweaning, fecal score of each pen (diarrhea score) was recorded every second day (days 1, 3, 5, 7, 9, 11, 13, and 15) based on a 1 to 5 scale (1: watery, 3: normal, and 5: firm), through visual observation of fresh feces in each pen (Guo et al., 2015). Observations were made based on the incidence of watery stool regardless of the number of pigs within a pen that showed signs of diarrhea. As an example, if both watery stool (1) and normal stool (3) were observed in a pen, the fecal score was recorded as 1. Fecal scores of 1 and 2 were classified as diarrhea, whereas scores of 3, 4, and 5 were classified as no diarrhea and diarrhea incidence was expressed as the proportion of pens showing diarrhea relative to the total number of pens in each group.

### Hematological evaluation

Blood samples were collected from 6 pens per treatment (1 barrow pen and 1 gilt pen from each group) via the jugular vein at the end of phase 1 and phase 2 (7 and 21 d postweaning, respectively). One pig representing a median BW of each pen was selected for blood sampling. Blood samples were used for hematological evaluation to measure immune-related cells including white blood cells, neutrophil, basophil, eosinophil, monocyte, red blood cells, platelets, and hemoglobin, using an automated hematological analyzer (CELL-Dyn 3200, Abbott Lab, Abbott Park, IL) following Mateo et al. (2006).

### Digestibility and retention of nitrogen

Three pens (2 barrow pens and 1 gilt pen) per treatment in group 1, 3 pens (1 barrow pen and 2 gilt pens) per treatment in group 2, and 5 pens (3 barrow pens and 2 gilt pens) per treatment in group 3 (*n* = 11 per treatment) were randomly selected and 1 pig representing a median BW of each pen was selected at the beginning of phase 3 and moved to individual metabolism crates (0.46 × 1.07 m) in the Texas Tech University Metabolism Building (New Deal, TX) for the 8-d metabolism trial. The metabolic crates allowed for separate collection of urine and feces. Pigs were fed the experimental diets twice daily (0700 and 1700 hours) in fixed amounts based on their initial BW (daily feed allowance, kg = 0.09 × BW^0.75^) for the first 4-d adaptation period and then a consecutive 4-d collection period. Pigs had free access to water.

The total fecal collection was initiated and terminated with the feeding of the first and last marked feed, which contained 0.25% chromic oxide as an indigestible marker. Collection of urine and feces samples was done for 4 d. The fecal collection was initiated when chromium color was observed in the feces and terminated when chromium color disappeared from the final meal. Urine collection was initiated when the test diets with chromium oxide were first provided and terminated when the final meal was offered. Urine samples were collected in plastic bottles with 15 mL HCl to prevent microbial growth (Stein et al., 2008; Kim et al., 2022; Choi et al., 2024), potentially leading to ammonia volatilization and underestimating urinary N output. The volume of urine was measured daily and 5% of each sample was taken as a daily subsample. All feces were collected and weighed at the end of each day during the 4-d collection period. Urine and feces were frozen at −20 °C immediately after collection. Fecal samples were freeze-dried for chemical analysis. Feed, feces, and urine samples were analyzed for dry matter (method 930.15), N (method 990.03), and crude ash (method 942.05) according to AOAC (2005). The apparent total tract digestibility of N and retention of N were calculated based on the following equations (Passos et al., 2015):


$$\begin{array}{lll} & Apparent\;total\;tract\;digestibility\;of\;N,\;\%\;=\;\; & \left[\left(N\;intake\;--\;N\;in\;feces\right)/N\;intake\right]\;\times \;100 \end{array}$$

$$\begin{array}{lll} & Retention\;of\;N,\;\%\;=\;\; & \left[\left(N\;intake\;--\;N\;in\;feces\;--\;N\;in\;urine\right)/N\;intake\right]\;\times \;100 \end{array}$$

where N intake is the total N intake (g), N in feces is the total fecal N output (g), and N in urine is the total urine N output (g) during the total collection period. The apparent total tract digestibility of DM was also calculated using the same equation. At the end of the 8-d period, pigs (1 pig per pen) were not returned to their originally assigned pens and thus growth performance data from these pigs were not included in the data from phase 3 to 6.

### Urine pH

Fresh urine samples were collected directly from the pigs on 25th day of phase 4 to measure urine pH. Two pens (1 barrow pen and 1 gilt pen) from each of the 0.00% and 1.00% of BA treatments were selected in groups 1, 2, and 3, respectively (*n* = 6 per treatment). For each pen, fresh urine samples were collected from 3 pigs and pooled to ensure homogeneity. Urine was collected between 0700 and 1000 hours. A person with a rod (3 m long) attached to a plastic bottle container (350 mL) collected urine from 3 pigs. Pigs were freely walkable within a pen during the entire collection period. For each pig, a new plastic bottle container was used. The pH was measured immediately in the urine after collection, using a calibrated pH meter (AB15 Basic, Thermo Fisher Scientific Inc., Waltham, MA).

### Aerial ammonia emission

From the second week of phase 3, 2 pens from groups 1, 2, and 3 from each of the 0.00% and 1.00% of BA treatments (*n* = 6 per treatment) were used to measure aerial ammonia emissions from the manure of pigs housed in an environmental chamber (Ji et al., 2006; Kim et al., 2006). All the 8 pigs in each pen were moved to a pen (1.2 × 2.4 m) in a ventilated environmental chamber (3.0 × 3.0 × 2.4 m) for 24 h during which aerial ammonia contents were measured. The chamber fan operated continuously at a constant speed (0.014 m^3^ air/s) throughout the experimental period. Two calibrated multigas monitors (Pac III and Miniwarn II, Draeger Safety, Inc., Pittsburgh, PA) with ammonia sensors were used to measure changes in ammonia levels over the 24-h period at 10 min intervals, and the average value from both devices was used as the final concentration. Feed intake of pigs during the 24-h period was measured. Initial and final BW were also measured before and after moving pigs between the original pen and the chamber. After the measurements, pigs were returned to their original pen. Feed intake and BW data obtained from 24 h period were included in the calculation of growth performance data.

### Carcass characteristics measurement

When pigs reached about 110 kg BW, 1 pig from each pen representing a median BW in groups 2 (4 pens) and 3 (7 pens) was moved to the Texas Tech Meat Laboratory to measure carcass characteristics including hot carcass weight, lean percentage, backfat thickness (at the 1st rib, last rib, and last lumbar vertebra), loin color, loin marbling score, and loin drip loss (*n* = 11 per treatment). Loin color was graded from 1 to 5 based on the NPPC Scoring System (1: pale and pinkish gray; 5: dark and purplish red; NPPC, 1991). The marbling score was also graded from 1 to 5 according to the NPPC Scoring System (1: devoid to practically devoid; 5: moderately abundant or greater; NPPC, 1999). Lean percentage was calculated by dividing the fat-free lean weight by hot carcass weight (Johnson et al., 2004). Fat-free lean weight (kg) was determined using the average of 2 equations: 1) fat-free lean weight, kg = 8.59 + (1.03 × hot carcass weight, kg)˗(0.863 × last rib backfat, mm) + (0.466 × loin eye area, cm^2^); 2) fat-free lean weight, kg = 23.6 + (1.11 × hot carcass weight, kg)˗(0.841 × last rib backfat, mm) following the previous studies (Burson and Berg, 2001; Yu et al., 2019).

### Statistical analysis

The data were analyzed using the MIXED procedure (SAS Inst., Cary, NC). The randomized complete block design was used with group as a block. The statistical model included dietary treatments as a fixed effect, with group as a random effect. The experimental unit was the pen for growth performance, diarrhea incidence, hematological evaluation, urine pH, ammonia emission, and carcass characteristics, whereas the experimental unit was the pig (representing a pen where a pig originated) for N digestibility and retention.

In the metabolism trial, 1 observation from a pig fed the diet containing antibiotics, 1 observation from a pig fed the diet with 0.00% BA, and 1 observation from a pig fed the diet with 0.50% BA for N digestibility and retention were removed as outliers as these observations deviated more than 1.5 fold of the interquartile range from the median value of their respective treatment groups. To determine the statistical significance of the expected mean difference of 15% at *P* < 0.05, using the coefficient of variation at 7.5% based on previous studies conducted using pigs with similar genetic backgrounds and research environments (Ji et al., 2006) and the power of test (1-beta) at 95% the power analysis indicated an 80%, the minimum number of replications for each treatment was 6 (Aaron and Hays, 2004).

The least squares mean for each treatment was calculated. The effects of increasing levels of BA in pig diets were determined using polynomial contrasts with coefficients calculated by the Proc IML procedure of SAS 9.4. The optimal level of BA intake on ADG was determined using a 1-slope broken-line analysis with the Proc NLMIXED procedure of SAS as described by Shen et al. (2012). Diarrhea incidence was analyzed using Proc GLIMMIX with beta distribution and logit link function and was expressed as the proportion of the number of pens of fecal score 2 or less divided by the total number of pens in each group. The optimal level of BA intake on G:F and loin muscle area was determined by a quadratic model with the Proc RSREG procedure of SAS.

The optimal level of BA for growth performance and carcass characteristics of pigs was calculated based on overall ADFI (1,427 g/d and 1,357 g/d, respectively). Ammonia levels during the collection period for each day per treatment (0.00% and 1.00% BA) were analyzed using the Proc GLM procedure of SAS to compare the slopes for 0.00% and 1.00% BA (Kim and Easter, 2001; Ji et al., 2006).

The statistical model used in the analysis is according to the following equation:


$$\begin{array}{l} y=a+b_{t}\times x_{t}+b_{s}\times x_{s}+e \end{array}$$

where *y* represents the response variable (ammonia emission, ppm), *a* is a common intercept (ammonia emission at 0 h in the chamber); *b*_t_ and *b*_s_ are the slopes for 0.00% and 1.00% BA on ammonia emission, respectively; *x*_t_ and *x*_s_ are hr in the chamber (hr) from 0.00% and 1.00% BA, respectively. The statistical significance and tendency were declared at *P* < 0.05 and 0.05 ≤ *P* < 0.10, respectively.

## Results

### Growth performance and diarrhea incidence

**Figure 1.**
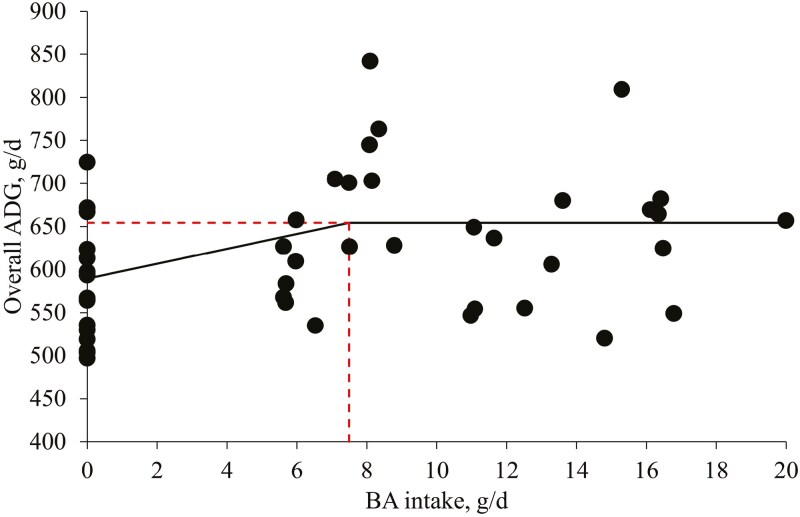
Overall ADG of pigs fed diets with increasing BA intake. The break point (a 1-slope broken-line analysis) was 7.5 g (standard error = 0.019; *P* < 0.05) of daily BA intake of pigs. The equation was: ADG, g/d = 654 − 8.65 × z1 (BA intake, g/d), *R*^2^ = 0.46 if BA intake is ≥ breakpoint, then z1 = 0. The optimal BA level was 0.53% for ADG of pigs calculated based on overall ADFI (1,427 g/d). The experimental unit was a pen and the number of observations was 45.

**Figure 2.**
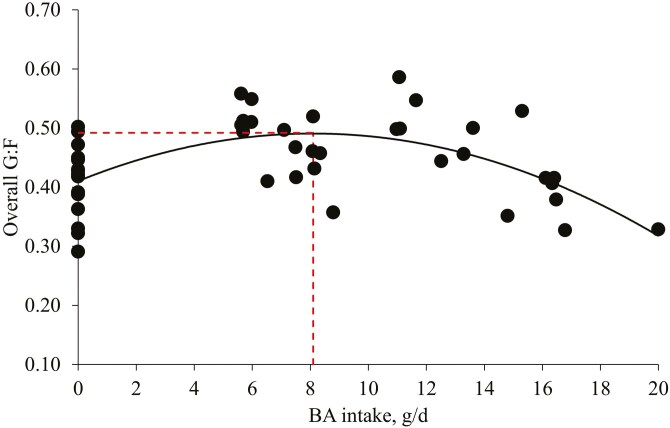
Overall gain-to-feed ratio (G:F) of pigs fed diets with increasing BA intake. Overall G:F = −0.001 × BA intake × BA intake + 0.020 × BA intake + 0.41 (Max: 0.49 at 8.1 g/d), *R*^2^ = 0.34; *P* value of the overall model: <0.001, *P* value of intercept: <0.001, and *P* value of BA intake × BA intake: <0.001; *P* value of BA intake: <0.001. The optimal BA level for G:F of pigs was 0.57% calculated based on overall ADFI (1,427 g/d). The experimental unit was a pen and the number of observations was 45.

The PC increased (*P* < 0.05) the final BW (110.7 kg) compared to no BA supplementation (96.1 kg). The PC increased (*P* < 0.05) the ADG during phases 3, 4, and 6 compared to no BA supplementation (Table 2). Increasing levels of BA increased (*P* < 0.05) ADG linearly during phases 3 and 5, and the overall period, and increased (*P* < 0.05) ADG quadratically during phase 4 and the overall period. Based on the broken-line analysis, increasing BA intake increased (*P* < 0.05) ADG during the overall period until BA intake was increased from 0.0 to 7.5 g/d of BA (Figure 1). The optimal level of BA for overall ADG was 0.53% calculated based on ADFI (1,427 g/d). The PC decreased (*P* < 0.05) the ADFI during phase 3 compared to no BA supplementation. Increasing levels of BA increased (*P* < 0.05) ADFI linearly during phase 3. The PC increased (*P* < 0.05) G:F during phases 1 to 4 and the overall period compared to no BA supplementation. Increasing levels of BA increased (*P* < 0.05) G:F linearly during phase 3 and the overall period and increased (*P* < 0.05) G:F quadratically during phase 4 and the overall period. Increasing BA intake increased G:F quadratically during the overall period (maximum at 8.1 g/d; Figure 2). The optimal level of BA on G:F during the overall period was 0.57% calculated based on ADFI (1,427 g/d). Diarrhea incidence was not affected by the PC or increasing levels of BA (Table 3).

**Table 2.** Growth performance of pigs fed diets with either antibiotics or increasing levels of BA^1^

		BA, %				*P* value		
Item	PC^2^	0.00	0.50	1.00	SEM	PC vs. 0.00	Linear^3^	Quadratic^4^
Body weight, kg								
Initial	5.9	5.9	5.9	6.0	0.5	0.924	0.940	0.990
Phase 1	6.9	6.8	6.8	6.7	0.5	0.866	0.961	0.961
Phase 2	11.4	10.8	10.9	10.9	0.7	0.522	0.883	0.907
Phase 3	20.8	17.8	19.8	20.0	1.7	0.027	0.103	0.436
Phase 4	62.9	49.1	58.1	53.1	5.0	<0.001	0.149	0.005
Phase 5	96.3	81.1	93.0	88.3	5.2	<0.001	0.047	0.009
Phase 6	110.7	96.1	107.7	103.3	4.5	<0.001	0.059	0.015
Average daily gain, kg/d								
Phase 1	0.142	0.119	0.117	0.108	0.014	0.169	0.505	0.823
Phase 2	0.319	0.283	0.294	0.295	0.017	0.143	0.612	0.813
Phase 3	0.450	0.337	0.423	0.435	0.060	<0.001	0.002	0.164
Phase 4	0.686	0.505	0.625	0.534	0.048	<0.001	0.393	<0.001
Phase 5	0.958	0.914	0.991	1.006	0.096	0.339	0.050	0.433
Phase 6	1.023	1.057	1.044	1.044	0.174	0.697	0.878	0.939
Overall	0.689	0.593	0.669	0.639	0.040	<0.001	0.039	0.007
Average daily feed intake, kg/d								
Phase 1	0.200	0.205	0.190	0.187	0.017	0.757	0.313	0.709
Phase 2	0.574	0.597	0.556	0.600	0.084	0.510	0.929	0.159
Phase 3	0.651	0.576	0.617	0.638	0.022	0.016	0.043	0.715
Phase 4	1.090	1.110	1.054	1.066	0.096	0.645	0.317	0.366
Phase 5	2.593	2.726	2.618	2.730	0.208	0.199	0.965	0.220
Phase 6	2.864	3.050	2.997	3.110	0.355	0.226	0.697	0.530
Overall	1.450	1.495	1.448	1.495	0.137	0.345	0.999	0.256
Gain-to-feed ratio								
Phase 1	0.71	0.58	0.62	0.58	0.08	0.016	0.911	0.383
Phase 2	0.59	0.49	0.56	0.51	0.06	0.010	0.634	0.069
Phase 3	0.70	0.58	0.70	0.69	0.10	0.030	0.048	0.190
Phase 4	0.64	0.47	0.61	0.53	0.08	<0.001	0.108	<0.001
Phase 5	0.37	0.33	0.38	0.37	0.02	0.111	0.115	0.111
Phase 6	0.35	0.36	0.34	0.34	0.03	0.852	0.523	0.789
Overall	0.48	0.40	0.47	0.44	0.03	<0.001	0.046	0.002

^1^The experimental unit was a pen, consisting of 8 pigs per pen, and each least squares mean represents 15 observations.

^2^PC = a basal diet with antibiotics (chlortetracycline at 100 mg/kg [450 mg/kg of Aureomycin 100] during phases 1 to 3, and tylosin at 44 mg/kg [200 mg/kg of Tylan 100] during phases 4 to 6).

^3^Linear = linear effect of increasing levels of BA.

^4^Quadratic = quadratic effect of increasing levels of BA.

**Table 3.** Diarrhea incidence of pigs fed diets with either antibiotics or increasing levels of BA^1^

		BA, %				*P* value		
Item	PC^2^	0.00	0.50	1.00	SEM	PC vs. 0.00	Linear^3^	Quadratic^4^
Phase 1 (days 0 to 7)	35.9	33.5	39.1	30.1	13.0	0.623	0.450	0.147
Phase 2 (days 7 to 15)	24.9	33.5	31.2	25.2	11.9	0.237	0.253	0.734
Overall (days 0 to 15)	29.7	32.9	31.1	27.0	13.4	0.556	0.289	0.787

^1^The experimental unit was a pen, consisting of 8 pigs per pen, and each least squares mean represents 15 observations.

^2^PC = a basal diet with antibiotics (chlortetracycline at 100 mg/kg [450 mg/kg of Aureomycin 100] during phases 1 to 3, and tylosin at 44 mg/kg [200 mg/kg of Tylan 100] during phases 4 to 6).

^3^Linear = linear effect of increasing levels of BA.

^4^Quadratic = quadratic effect of increasing levels of BA.

### Hematological evaluation

Increasing levels of BA tended to decrease (*P* = 0.056) monocyte contents linearly in blood and increased (*P* < 0.05) red blood cells, hemoglobin, and hematocrit contents quadratically in blood on 7 d postweaning (Table 4). On 21 d postweaning, increasing levels of BA increased (*P* < 0.05) eosinophil contents both linearly and quadratically and increased (*P* < 0.05) mean corpuscular hemoglobin quadratically in blood. However, white blood cells, neutrophils, lymphocytes, and mean corpuscular volume in blood were not affected by the PC or increasing levels of BA.

**Table 4.** Hematological evaluation in pigs fed diets with either antibiotics or increasing levels of BA (as-is basis)^1^^,^^2^

		BA, %				*P* value		
Item	PC^3^	0.00	0.50	1.00	SEM	PC vs. 0.00	Linear^4^	Quadratic^5^
7 d postweaning (28 d of age)								
White blood cell, 10^3^ cell/uL	10.9	9.7	11.1	8.6	4.6	0.500	0.535	0.209
Neutrophil, 10^3^ cell/uL	7.3	6.6	6.4	5.5	2.0	0.628	0.450	0.765
Lymphocyte, 10^3^ cell/uL	4.4	5.3	5.4	4.5	4.3	0.305	0.369	0.498
Monocyte, 10^3^ cell/uL	0.28	0.42	0.22	0.22	0.16	0.170	0.056	0.250
Eosinophil, 10^3^ cell/uL	0.08	0.11	0.08	0.10	0.02	0.240	0.646	0.443
Basophil, 10^3^ cell/uL	3.76	3.42	3.46	3.45	3.42	0.001	0.702	0.706
Red blood cell, 10^6^ cell/uL	9.29	8.42	9.59	7.88	2.4	0.165	0.375	0.012
Hemoglobin, g/dL	23.9	21.6	24.4	20.4	9.9	0.164	0.473	0.024
Hematocrit, %	41.7	39.5	43.8	37.1	6.5	0.462	0.413	0.042
Mean corpuscular volume, fL	35.9	33.5	34.6	33.3	18.3	0.243	0.928	0.507
Mean corpuscular hemoglobin, pg	27.9	26.4	27.0	26.3	7.4	0.140	0.905	0.428
21 d postweaning (42 d of age)								
White blood cell, 10^3^ cell/uL	12.9	10.7	11.6	10.4	5.9	0.392	0.895	0.597
Neutrophil, 10^3^ cell/uL	7.8	9.4	7.8	7.4	3.0	0.403	0.309	0.729
Lymphocyte, 10^3^ cell/uL	6.7	4.8	5.9	4.6	5.0	0.315	0.891	0.450
Monocyte, 10^3^ cell/uL	0.5	0.6	0.5	0.5	0.3	0.485	0.611	0.295
Eosinophil, 10^3^ cell/uL	0.2	0.2	0.2	0.4	0.1	0.832	0.035	0.039
Basophil, 10^3^ cell/uL	3.1	3.3	2.7	3.1	3.0	0.706	0.799	0.232
Red blood cell, 10^6^ cell/uL	9.4	9.6	8.5	9.7	2.9	0.807	0.915	0.185
Hemoglobin, g/dL	22.1	23.0	19.8	22.2	10.6	0.740	0.759	0.253
Hematocrit, %	43.3	42.7	42.9	42.5	11.0	0.679	0.896	0.811
Mean corpuscular volume, fL	35.1	35.1	36.7	33.0	14.8	0.987	0.306	0.143
Mean corpuscular hemoglobin, pg	27.7	27.3	28.5	26.8	10.1	0.585	0.585	0.047

^1^The experimental unit was a pen (1 pig representing the median BW was selected in each pen), and each least squares mean represents 6 observations.

^2^Hematological analysis was done using an automated hematology analyzer (CELL-Dyn 3200, Abbott Lab, Abbot Park, IL).

^3^PC = a basal diet with antibiotics (chlortetracycline at 100 mg/kg [450 mg/kg of Aureomycin 100] during phases 1 to 3, and tylosin at 44 mg/kg [200 mg/kg of Tylan 100] during phases 4 to 6).

^4^Linear = linear effect of increasing levels of BA.

^5^Quadratic = quadratic effect of increasing levels of BA.

### Digestibility and retention of nitrogen

Increasing levels of BA tended to increase (*P* = 0.096) N digestibility and increased (*P* < 0.05) N retention linearly (Table 5). However, fecal N output and urinary N output were not affected by the PC or increasing levels of BA.

**Table 5.** Nitrogen balance in pigs fed diets with either antibiotics or increasing levels of BA^1^^,^^2^

		BA, %				*P* value		
Item^3^	PC^4^	0.00	0.50	1.00	SEM	PC vs. 0.00	Linear^5^	Quadratic^6^
Initial BW, kg	12.6	11.9	12.2	11.9	0.7	0.464	0.962	0.704
ADFI, g/d	602	575	587	576	26	0.461	0.958	0.715
Dry matter								
Intake, g/d	540	515	526	517	23	0.461	0.958	0.715
Fecal output, g/d	78	81	77	72	10	0.645	0.240	0.908
Digestibility, %	85.6	84.1	85.3	86.0	1.8	0.309	0.177	0.877
Nitrogen								
Intake, g/d	18.3	17.4	17.8	17.5	0.8	0.451	0.954	0.695
Fecal output, g/d	3.4	3.6	3.3	3.0	0.5	0.619	0.124	0.907
Urine output, g/d	4.6	4.2	4.1	3.3	0.6	0.584	0.306	0.621
Digestibility, %	81.3	79.1	81.5	82.6	2.8	0.308	0.096	0.710
Retention, %	55.6	54.2	57.9	63.4	3.4	0.764	0.046	0.812

^1^The experimental unit was a pig, and each least squares mean represents 10 observations, except for 1.00% BA, which have 11 observations.

^2^Pigs were housed individually in metabolism crates for this study.

^3^BW = body weight; ADFI = average daily feed intake.

^4^PC = a basal diet with antibiotics (chlortetracycline at 100 mg/kg [450 mg/kg of Aureomycin 100] during phases 1 to 3, and tylosin at 44 mg/kg [200 mg/kg of Tylan 100] during phases 4 to 6).

^5^Linear = linear effect of increasing levels of BA.

^6^Quadratic = quadratic effect of increasing levels of BA.

### Acidification of urine and aerial ammonia emission in chamber

**Figure 3.**
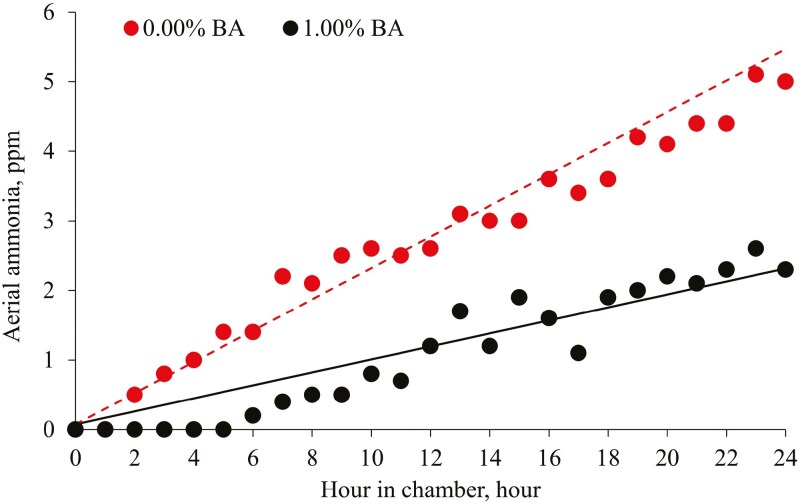
Concentration of aerial ammonia in the environmental chamber produced from the manure in pigs fed 0.00% or 1.00% BA supplementation in the 24 h collection period (indicated as 0 to 24 h on the x-axis). The changes in aerial ammonia concentrations, ppm were as follows: (Ammonia, ppm)_0.00% BA_ = 0.225 × hour + 0.076, *R*^2^ = 0.96 and (Ammonia, ppm)_1.00% BA_ = 0.093 × hour + 0.076, *R*^2^ = 0.96. *P* value of the overall model: <0.001, *P* value of the intercept: 0.399, and *P* values for the slopes [*x*_t_ (0.00% BA) and *x*_s_ (1.00% BA)] were <0.001 and <0.001, respectively. *P* value for the contrast between the slopes of 0.00% and 1.00% BA was <0.001. The experimental unit was a pen and the number of observations was 6 per treatment.

BA supplementation at 1% decreased (*P* < 0.05) urine pH compared to no BA supplementation (Table 6). BA supplementation at 1.00% decreased (*P* < 0.05) urine pH and the average aerial ammonia emission from manure compared to no BA supplementation. BA supplementation at 1.00% decreased (*P* < 0.05) the rate of change in aerial ammonia emission compared to no BA supplementation in the 24 h collection period (Figure 3).

**Table 6.** Urine pH and aerial ammonia emission in pigs fed diets with 0.00 or 1.00% BA^1^^,^^2^

	BA, %			
Item	0.00	1.00	SEM	*P* value
Urine pH	7.37	6.21	0.25	0.008
Hour in chamber, ppm				
24 h average	2.87	1.10	0.53	<0.001
Last 3 h	5.33	2.47	1.03	<0.001
Last 5 h	5.00	2.38	1.02	<0.001
Last 7 h	4.78	2.27	0.93	0.001
Last 10 h	4.43	2.03	0.84	0.001

^1^The experimental unit was a pen and each least squares mean represents 6 observations for urine pH and ammonia emission.

^2^Pigs were housed in a pen in an environmental chamber with an odor sensor for 24 h period for this study.

### Carcass characteristics measurement

**Figure 4.**
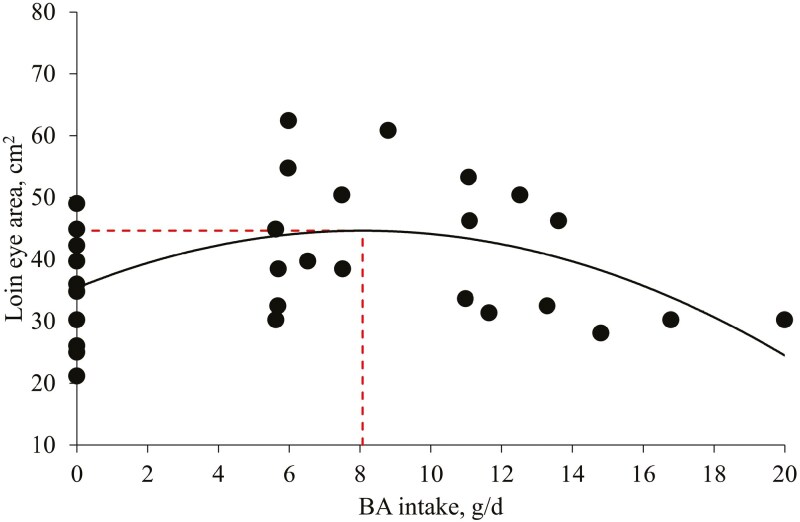
Loin eye area in pigs fed diets with increasing BA intake. Loin eye area, cm^2^ = −0.14 × BA intake × BA intake + 2.29 × BA intake + 35.4 (Max: 44.7 cm^2^ at 8.1 g/d), *R*^2^ = 0.22; *P* value of the overall model: <0.05, *P* value of the intercept: <0.05, and *P* value of BA intake × BA intake: <0.05; *P* value of BA intake: <0.05. The optimal BA level for the loin eye area in pigs was 0.59% calculated based on overall ADFI (1,357 g/d). The experimental unit was a pen (1 pig representing the median BW was selected in each pen) and the number of observations was 33.

The PC increased (*P* < 0.05) shrink weight, hot carcass weight, and first rib backfat compared to no BA supplementation (Table 7). Increasing levels of BA decreased (*P* < 0.05) loin color and marbling score linearly and increased (*P* < 0.05) the loin eye area quadratically. Increasing BA intake increased (*P* < 0.05) the loin eye area quadratically (maximum at 8.1 g/d; Figure 4). The optimal level of BA on the loin eye area was 0.59% calculated based on overall ADFI (1,357 g/d).

**Table 7.** Carcass characteristics of pigs fed diets with either antibiotics or increasing levels of BA^1^^,^^2^

		BA, %				*P* value		
Item	PC^3^	0.00	0.50	1.00	SEM	PC vs. 0.00	Linear^4^	Quadratic^5^
Shrink weight, kg	105.3	93.7	101.3	97.3	5.4	0.004	0.362	0.093
Hot carcass weight, kg	80.4	70.9	75.7	73.8	2.1	0.002	0.331	0.209
Dressing, %	76.5	75.9	74.5	75.7	4.0	0.541	0.827	0.133
Backfat thickness, mm								
First rib backfat	22.4	17.3	18.8	18.6	1.9	0.001	0.372	0.505
Last rib backfat	19.1	16.9	17.3	16.7	3.2	0.134	0.867	0.717
Last lumbar vertebra backfat	15.7	13.8	12.3	13.4	0.9	0.089	0.736	0.195
Loin eye area, cm^2^	43.41	35.34	45.26	38.23	3.34	0.088	0.545	0.045
Loin color^6^	3.00	3.09	2.90	2.40	0.21	0.746	0.020	0.542
Marbling score^7^	2.01	2.01	1.52	1.22	0.28	1.000	0.028	0.752
Lean %^8^	54.0	54.9	55.6	55.0	1.74	0.414	0.861	0.479
Drip loss, %	2.45	2.70	3.51	3.64	0.60	0.712	0.191	0.588

^1^The experimental unit was a pen (1 pig representing the median BW was selected in each pen), and each least squares mean represents 11 observations.

^2^Pigs were moved to Texas Tech Meat Lab and euthanized after overnight fasting.

^3^PC = a basal diet with antibiotics (chlortetracycline at 100 mg/kg [450 mg/kg of Aureomycin 100] during phases 1 to 3, and tylosin at 44 mg/kg [200 mg/kg of Tylan 100] during phases 4 to 6).

^4^Linear = linear effect of increasing levels of BA.

^5^Quadratic = quadratic effect of increasing levels of BA.

^6^NPPC Scoring System (1: pale, pinkish gray; 5: dark, purplish red).

^7^NPPC Scoring System (1: devoid to practically devoid; 5: moderately abundant or greater).

^8^Lean, % = fat-free lean weight (kg)/hot carcass weight (kg) × 100; fat-free lean weight (kg) was determined using the average of 2 equations: 1) equation 1, kg = 8.59 + (1.03 × hot carcass weight, kg) − (0.863 × last rib backfat, mm) + (0.466 × loin eye area, cm^2^); 2) equation 2, kg = 23.6 + (1.11 × hot carcass weight, kg) − (0.841 × last rib backfat, mm).

## Discussion

Similar to the results of this study, previous studies showed that N digestibility and retention are improved in pigs when they consume BA (Kluge et al., 2006; Diao et al., 2014). One possible explanation is that BA supplementation reduces the pH of digesta in the stomach (Chen et al., 2017; Perić et al., 2023), thereby enhancing protein denaturation and activation of pepsinogen. The reduction of pH in jejunal digesta caused by BA (Chen et al., 2017; Perić et al., 2023) can also modulate the luminal microbiota of pigs, inhibiting the growth of ammonia-producing bacteria such as *E. coli* (Guggenbuhl et al., 2007; Diao et al., 2014; Perić et al., 2023), whereas increasing the growth of *Lactobacillus* spp. (Chen et al., 2017). This modulation may further improve intestinal health (Diao et al., 2014) and enhance N utilization (Bühler et al., 2006; Humphrey et al., 2022).

The effects of organic acids on ammonia-producing bacteria, however, are not solely attributed to the reduction of digesta pH in the small intestine (Kovanda et al., 2019). Some previous studies reported that BA supplementation did not alter gastric or jejunal pH but still decreased ammonia-producing bacteria, such as *E. coli*, in the intestine of nursery pigs (Kluge et al., 2006; Diao et al., 2014). A possible explanation is that BA, as a weak organic acid, predominantly exists in its non-ionized form in the digesta of pigs (Kluge et al., 2006). The non-ionized form of BA is highly lipid-soluble, allowing BA to penetrate bacterial membrane (Park et al., 2001), particularly affecting Gram-negative bacteria. A potential reason BA affects Gram-negative bacteria like *Escherichia coli* is that lipid-soluble BA can interact with the lipid-soluble LPS in the bacterial membrane, disrupting bacterial activity (Walter and Gutknecht, 1984). Thus, BA can effectively reduce ammonia-producing bacteria, which may enhance N digestibility and retention. Previous studies reported that BA reduces urinary N excretion in pigs (Murphy et al., 2011; Humphrey et al., 2022), which may also enhance N retention in this study. The reduction in urinary N excretion could result from either increased protein synthesis or decreased protein turnover in pigs, helping to reduce excess amino acids in the body and, consequently, N excretion.

The antimicrobial effects of BA in reducing ammonia-producing Gram-negative bacteria, such as *Escherichia coli* in the digesta of pigs (Diao et al., 2014), contribute to lowering ammonia-N content (Halas et al., 2010; Diao et al., 2014) and may also decrease the incidence of postweaning diarrhea in pigs (Halas et al., 2010; Silveira et al., 2018), supporting their growth. In this study, however, BA supplementation did not affect diarrhea incidence during the postweaning period. This discrepancy may be due to experimental conditions, particularly whether the pigs were challenged with *Escherichia coli* (Halas et al., 2010; Silveira et al., 2018). Under challenge conditions, the antimicrobial effects of BA on digesta and diarrhea could be more pronounced, as there would be an increased presence of *E. coli* for BA to act upon (Qi et al., 2024). This could explain the greater reduction in diarrhea observed in previous studies (Halas et al., 2010; Silveira et al., 2018).

In this study, the inclusion of chlortetracycline and tylosin in the diets improved BW gain by 14 kg compared to pigs fed diets without antibiotics, which is consistent with findings from previous studies and reviews (Stahly et al., 1980; Zimmerman, 1986; Cromwell, 2002). However, growing concerns regarding antimicrobial resistance have led to the ban or restriction of antimicrobial growth promoters in several countries (Casewell et al., 2003; Tiseo et al., 2020).

In this study, increasing levels of BA linearly improved N digestibility and retention, which is known to have positive effects on growth, whereas quadratic responses were observed in growth performance, with the maximum at 0.53% to 0.55% BA. This study focused on discussing quadratic response to BA, based on previous literature (Nair, 2001; Shu et al., 2016) supporting the hypothesis and allowing the estimation of the biological optimum level. This also aligns with the finding from a previous study, which reported 0.60% BA as the optimal level for BW gain during the nursery phase (Choi and Kim, 2024). The quadratic response may be due to increased BA content in the liver and kidneys (Shu et al., 2016), resulting in systemic acidosis at high doses of BA (Nair, 2001). High BA supplementation over 2.50% in diets also reduced red and white blood cell counts, both of which are crucial for oxygen transport and immune response, and increased oxidative stress in the hepatic cells of pigs, whereas no negative impacts were observed at 0.50% BA in pig feeds (Shu et al., 2016). In this study, increasing levels of BA up to 1.00% also showed quadratic changes in blood parameters, including eosinophil levels and mean corpuscular hemoglobin after the 21-d postweaning period. Thus, an appropriate BA level could benefit the growth performance of pigs by improving N digestibility and retention.

BA is absorbed in the small intestine (Kristensen et al., 2009) and transported to the liver, where it conjugates with glycine to form hippuric acid (Bühler et al., 2006), which is subsequently excreted in urine (Choi et al., 2023; Yoo et al., 2024). Previous studies reported that increased excretion of hippuric acid by BA reduces the pH of urine and manure (Bühler et al., 2006; Choi et al., 2023). Additionally, 1.00% BA supplementation decreased urine pH from 7.6 to 6.5 in pigs (Bühler et al., 2006; Kluge et al., 2006), which is consistent with the results of this study. A reduction of approximately 1 pH unit in urine represents a tenfold difference in acidity (Covington et al., 1985), which can also contribute to reducing the production of ammonia and odorous compounds, such as indole, in pig manure (Murphy et al., 2011; Yoo et al., 2024). A possible reason for the reduction in ammonia emissions from urine and manure is that a lower manure pH retains N in the ammonium form, thereby limiting its conversion to volatile ammonia gas (Ji et al., 2006; Kim et al., 2022; Le Dinh et al., 2022). Additionally, the reduced pH in urine and manure can inhibit urease activity (Panja and Adams, 2019), an enzyme responsible for converting urea into ammonia, further reducing ammonia formation. It is important to distinguish between ammonia production, which can originate from amino acid deamination, microbial metabolism, or urease-mediated urea hydrolysis, and ammonia volatilization, which refers to the release of ammonia gas into the air. Reduced urinary N excretion would also contribute to lower ammonia production (Murphy et al., 2011; Humphrey et al., 2022); however, the degree of improvement observed in this study suggests that the primary factor contributing to reduced ammonia emission is the lowered urinary pH, which reduces the conversion of ammonium ion into ammonia gas. Consequently, diets containing BA can improve air quality in pig barns and mitigate their environmental impacts (De Vries and Melse, 2017).

In this study, BA supplementation, with optimal effects observed at 0.53% to 0.59% BA, improved growth performance, which may explain the observed increase in the loin eye area, as an increase in muscle area partially reflects the amount of muscle growth contributing to BW gain. The loin eye area represents the total cross-sectional area of the loin muscle, including both lean tissue and intramuscular fat. In agreement with the results of this study, BA supplementation at 1.00% in pig feeds reduced the marbling score and influenced loin color (Cheng et al., 2017). The marbling score reflects the amount and distribution of intramuscular fat in pork, indicating that BA supplementation may shift nutrient partitioning, favoring protein deposition over lipid deposition in pigs (Faucitano et al., 2005). Thus, BA could enhance lean meat production by improving N retention, potentially explaining the reduced marbling score observed in this study.

BA supplementation in feeds increased BA and hippuric acid contents in the blood of pigs (Kristensen et al., 2009), which could reduce pH in meat (Brewer et al., 2001). The reduced pH in meat may lower loin color score, as acidic compounds increase protein denaturation in meat, potentially contributing to the pale color and softer texture observed in this study (Brewer et al., 2001), indicating that a high level of BA supplementation could cause pale, soft, exudative meat. Studies in growing pigs, however, reported no effects on growth performance or carcass characteristics with up to 1.00% BA supplementation (Morel et al., 2019; O’Meara et al., 2020). These discrepancies may result from differences in the growth stage of pigs. The nursery phase is the most critical stage in the life of a pig, as nursery pigs have immature digestive systems that are highly susceptible to various stress factors caused by weaning (Duarte and Kim, 2022). As a result, the effects of BA on the growth of nursery pigs may be more pronounced than in growing pigs (Gaffield et al., 2024). The positive effects of BA during the nursery phase could have contributed to the improved growth performance and carcass characteristics of pigs observed in this study.

In conclusion, increasing levels of BA linearly improved N digestibility and retention and showed quadratic increases in the growth performance and carcass characteristics of pigs, suggesting that the beneficial effects of BA can be maximized within a range of 0.53% to 0.59% (7.5 to 8.1 g/d of BA), based on overall ADFI, for the growth and carcass characteristics of pigs. BA supplementation at 1.00% also increased urine acidification and reduced aerial ammonia emissions when fed to pigs. These results highlight that BA supplementation within the range of 0.5% to 0.6% provides optimal efficiency for N utilization, pig growth, and pigs from weaning to market.

## Abbreviations:

ADFI: average daily feed intake
ADG: average daily gain
BA: benzoic acid
BW: body weight
G:F: gain-to-feed ratio
N: nitrogen
PC: antibiotics treatment
